# Supplementary material for: CaClust: linking genotype to transcriptional heterogeneity of follicular lymphoma using BCR and exomic variants
Source: Genome Biol. 2024 Nov 5;25:286. doi: 10.1186/s13059-024-03417-1 (PMC11536712; doi:10.1186/s13059-024-03417-1)
Supplement: Supplementary file 1 — Additional file 1: Supplementary Figures S1-S14. [file 13059_2024_3417_MOESM1_ESM.pdf]

Supplementary Figures

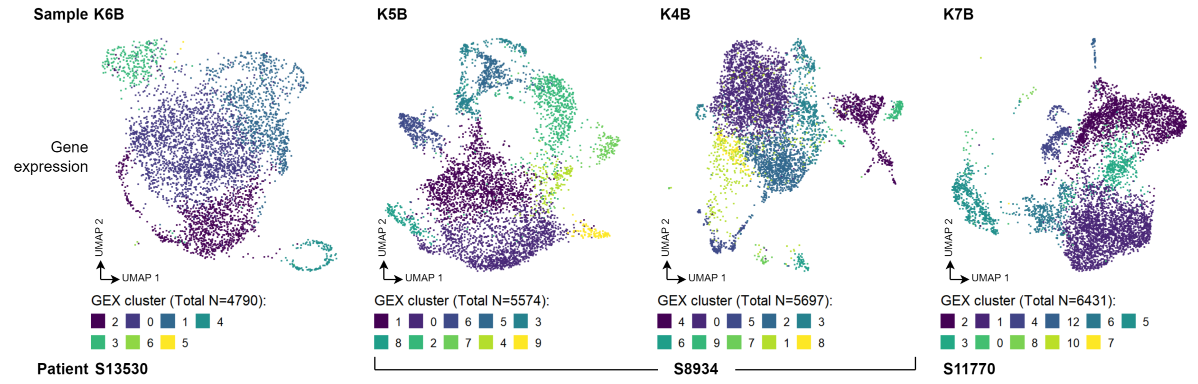

Fig. S1: Clustering of cells by gene expression in each sample separately. Gene expression clusters were obtained by PCA dimensionality reduction to 30 dimensions and next Leiden clustering on the reduced profiles with resolution parameter of 0.3m using Seurat package.

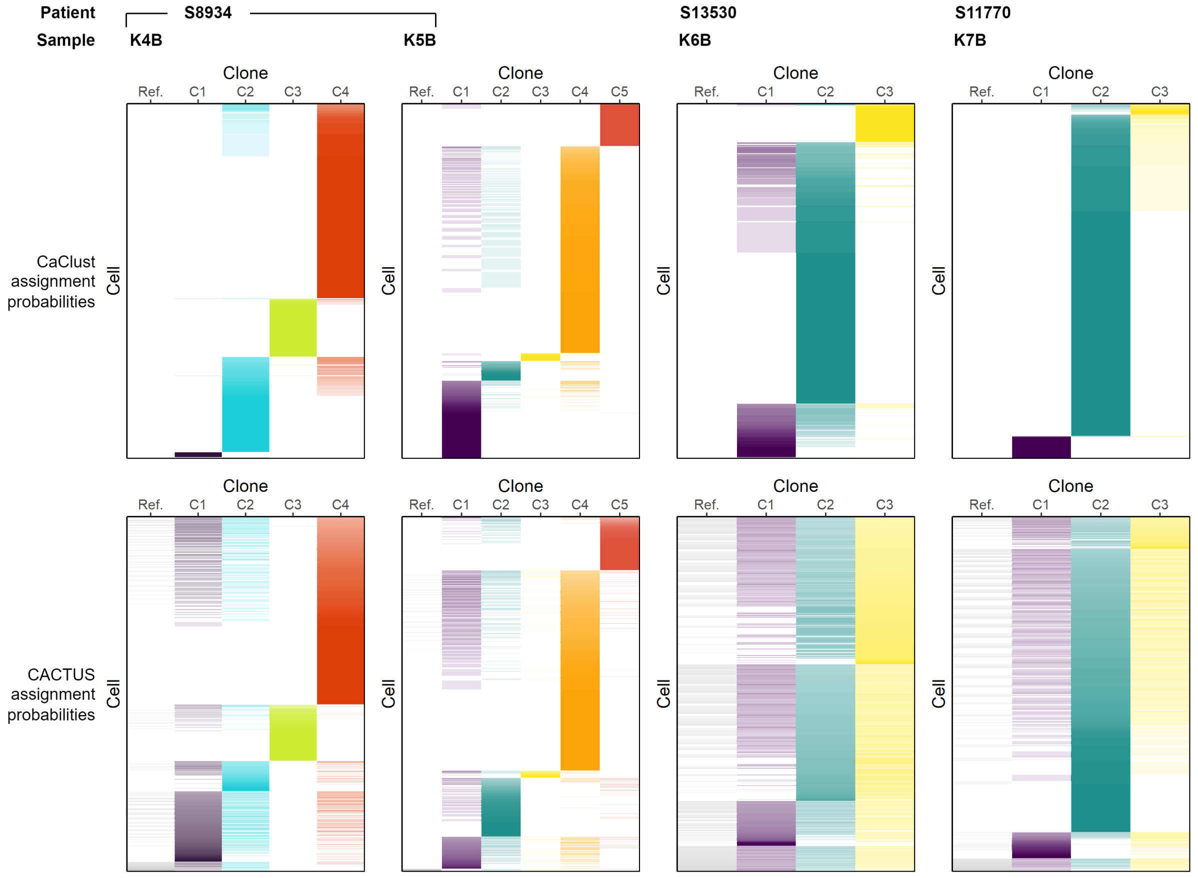

Fig. S2: Heatmaps of assignment probabilities of cells to clones in all samples for CaClust and CACTUS. Cells order is not the same between methods. Clones are not shared between samples. Clone genotypes between methods are not identical for each sample; additionaly, CaClust clones C1 and C2 in each of the samples K4B and K5B are the counterparts of CACTUS clones C2 and C1 in those samples (resulting from the probabilistic nature of genotype correction in the methods).

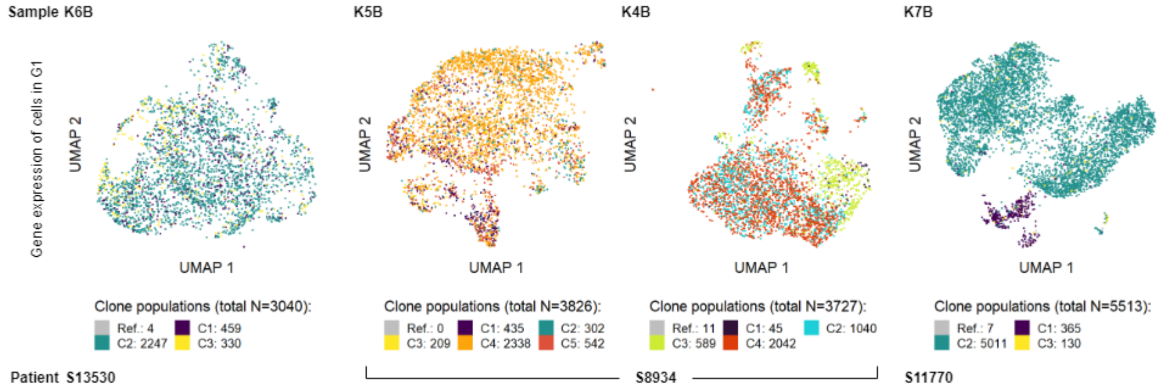

Fig. S3: UMAP plots of expression of cells in phase G1 in clones of each sample. Cells are colour coded by clone assignment and their numbers in each clone are provided in the legend. The clones are not shared between samples. The samples are ordered by apparent influence of the clone genotypes on their phenotypic heterogeneity.

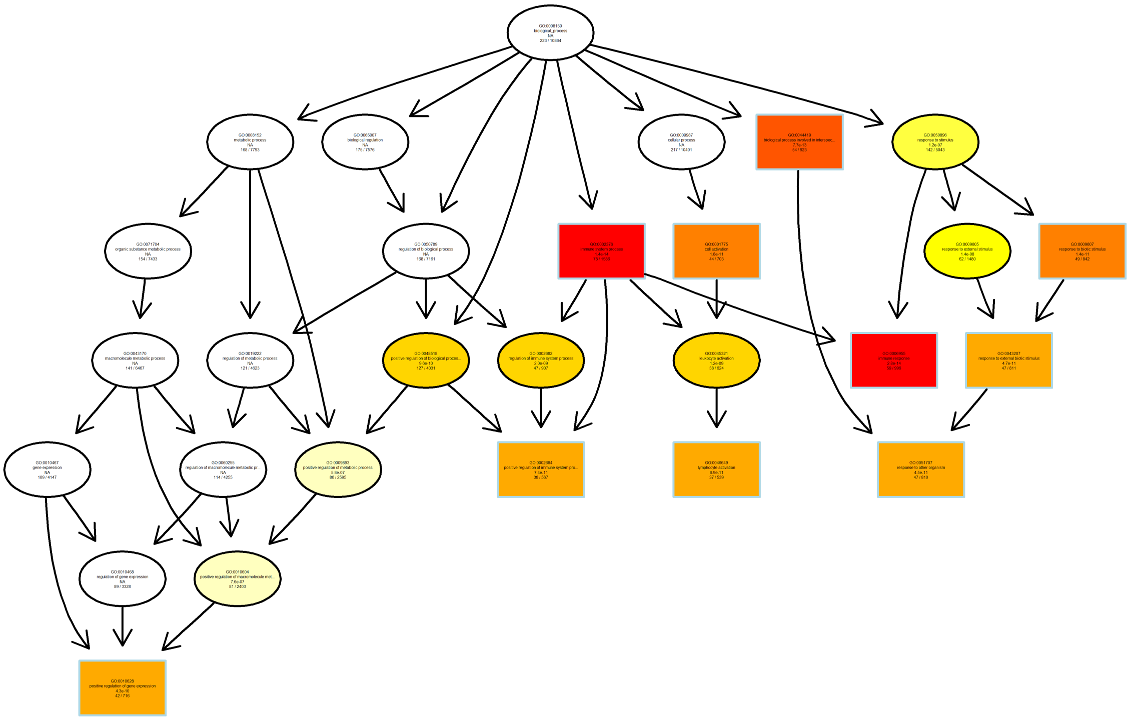

Fig. S4: Enrichment of Gene Ontology terms in top 250 most variably expressed genes of sample K5B. Top 10 biological process found by Fisher's exact test are shown.

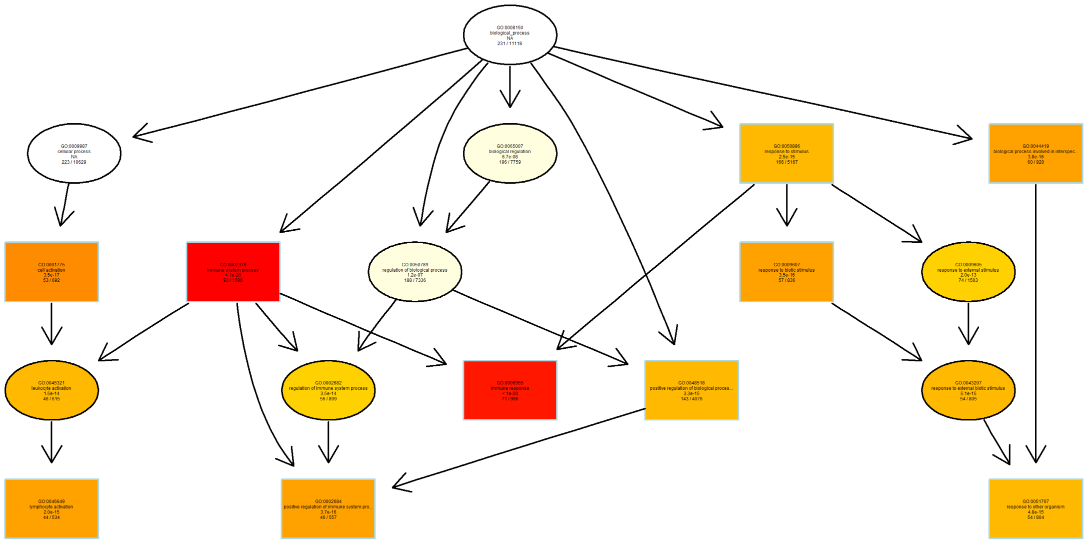

Fig. S5: Enrichment of Gene Ontology terms in top 250 most variably expressed genes of sample K6B. Top 10 biological process found by Fisher's exact test are shown.

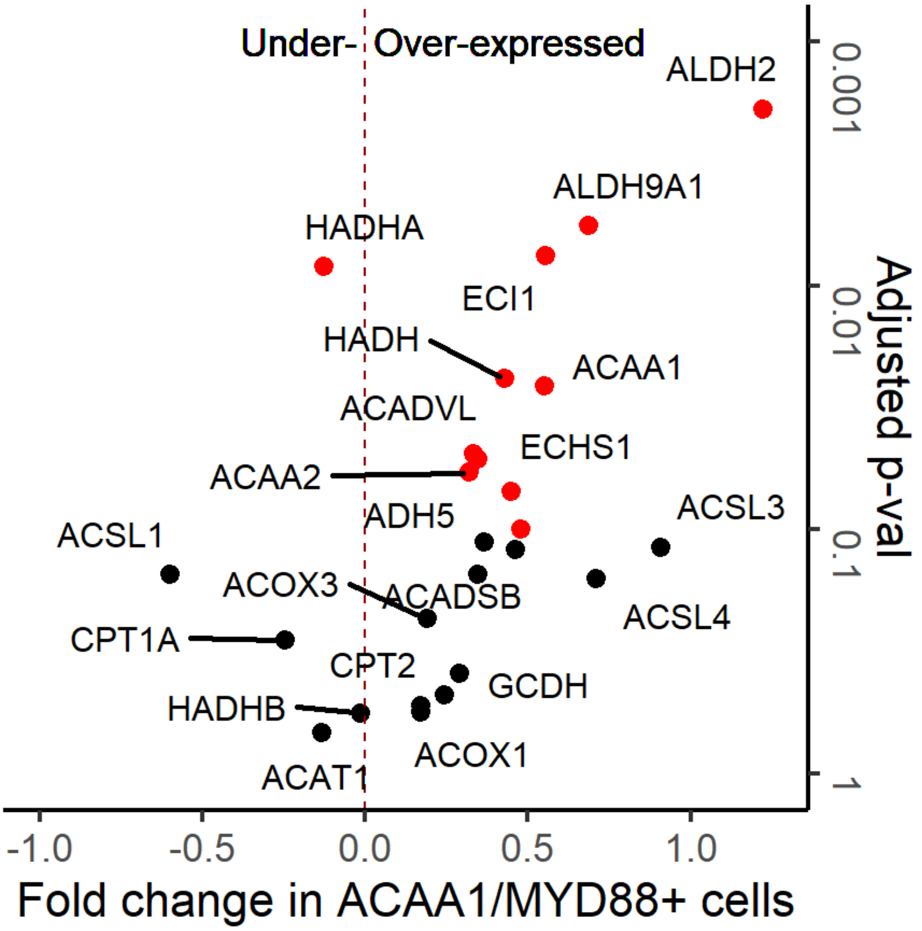

Fig. S6: Enrichment of betaoxidation pathway genes in K7B clone C2. Genes with $p_{adj} < 0.1$ are highlighted.

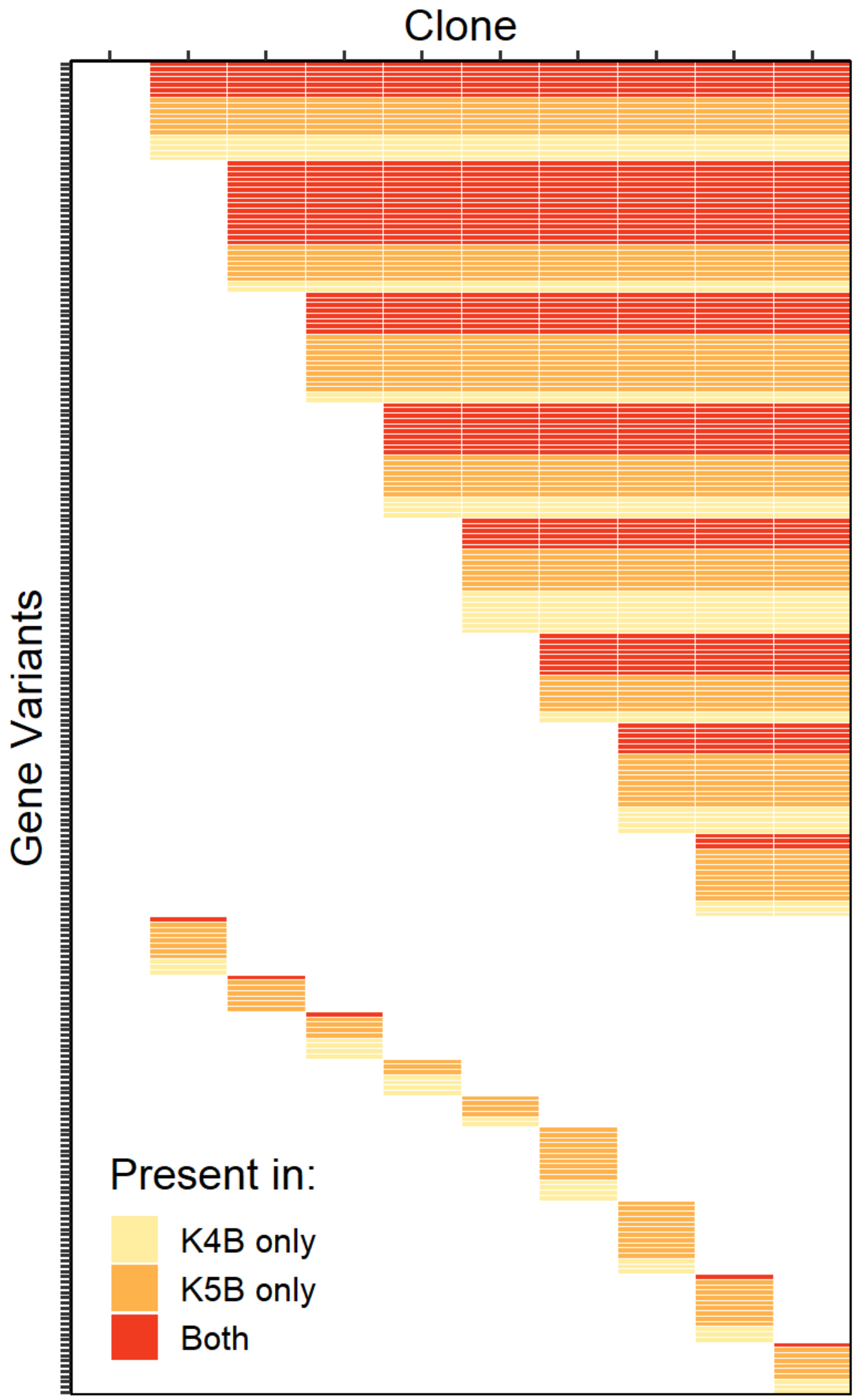

Fig. S7: Phylogeny of the joint K4B and K5B samples obtained with CANOPY for 9 tumour clones and 1 reference clone. Positions in red were found mutated in WES of both samples, positions in orange were only mutated in sample K5B, and positions in yellow were only mutated in sample K4B.

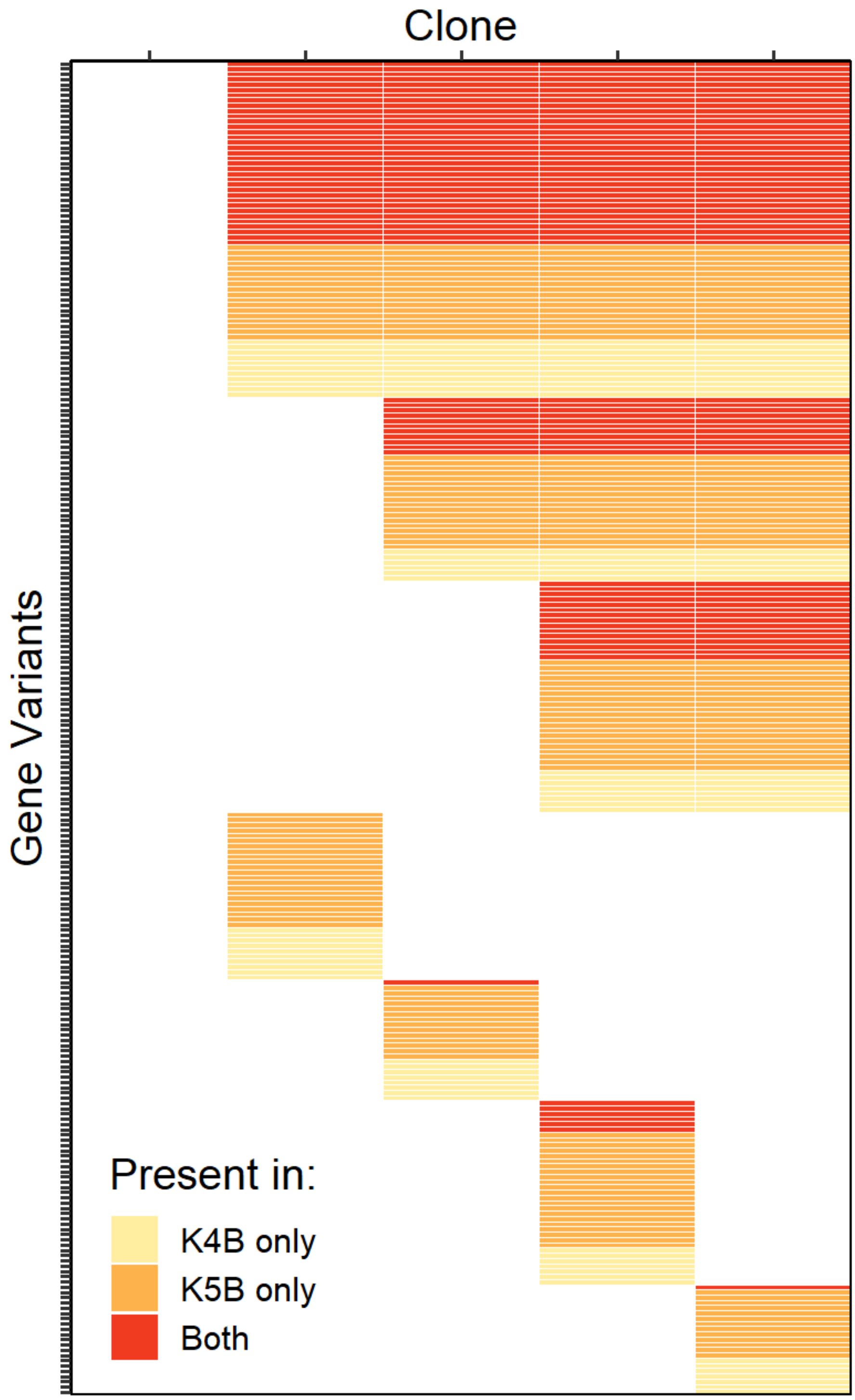

Fig. S8: Phylogeny of the joint K4B and K5B samples obtained with CANOPY for 4 tumour clones and 1 reference clone. Positions in red were found mutated in WES of both samples, positions in orange were only mutated in sample K5B, and positions in yellow were only mutated in sample K4B.

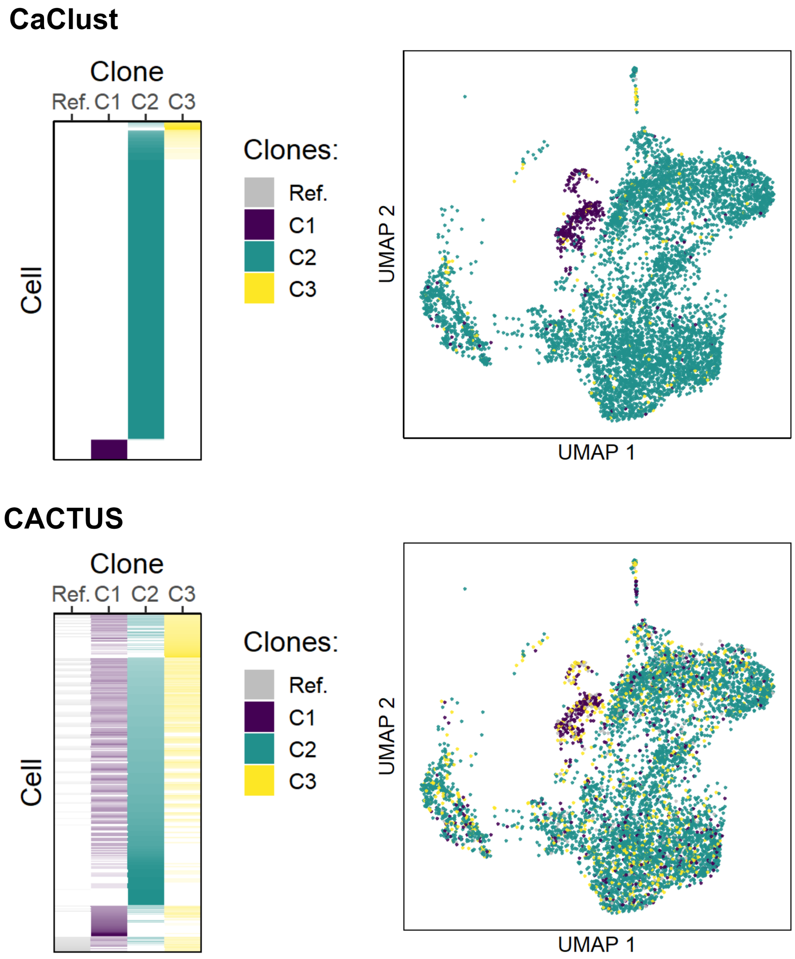

Fig. S9: Comparison of CaClust and CACTUS results assignment confidence and mapping to gene expression in sample K7B.

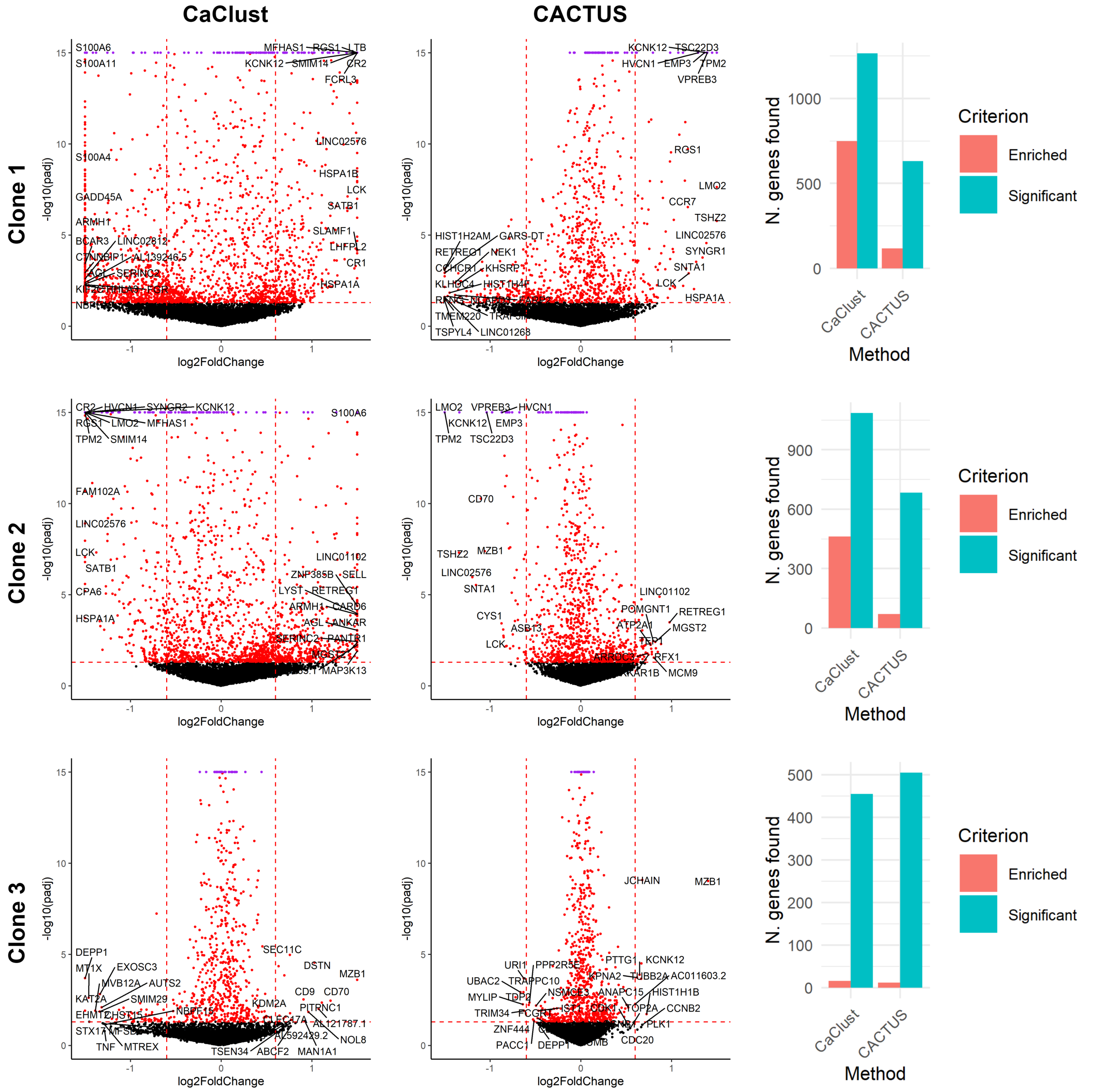

Fig. S10: Comparison of DE analysis of CaClust and CACTUS results for sample K7B. First two columns contain volcano plots for clones obtained with CaClust and CACTUS respectively, the last column contains the comparison of the number of genes identified in each method that are: significant ($p_{adj} < 0.05$), enriched ($p_{adj} < 0.05$ and absolute log2 fold change > 0.6).

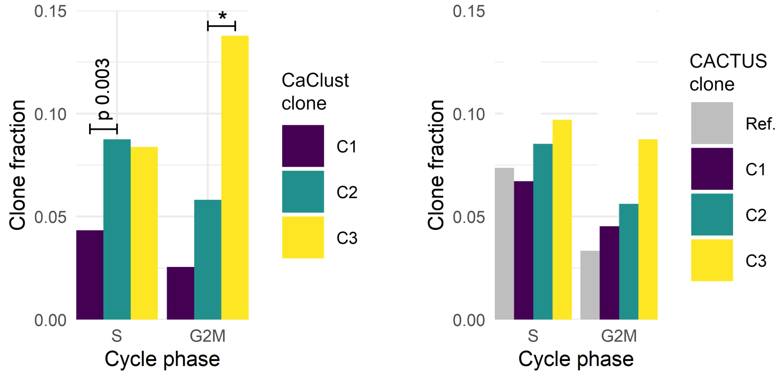

Fig. S11: Comparison of cell cycle distribution of CaClust and CACTUS clones in sample K7B.

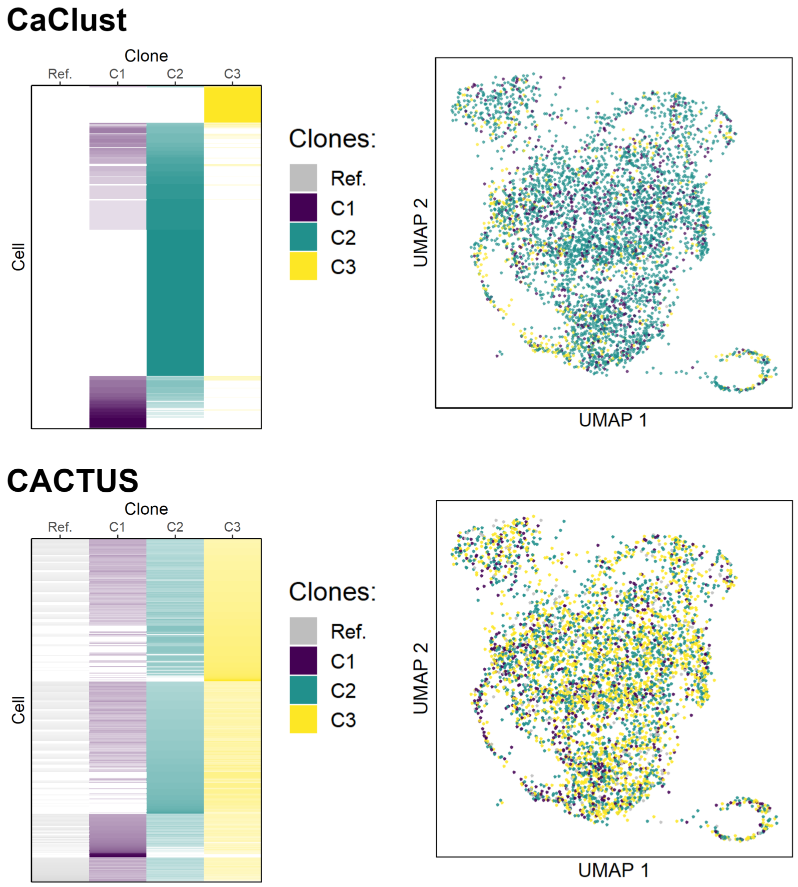

Fig. S12: Comparison of CaClust and CACTUS results assignment confidence and mapping to gene expression in sample K6B.

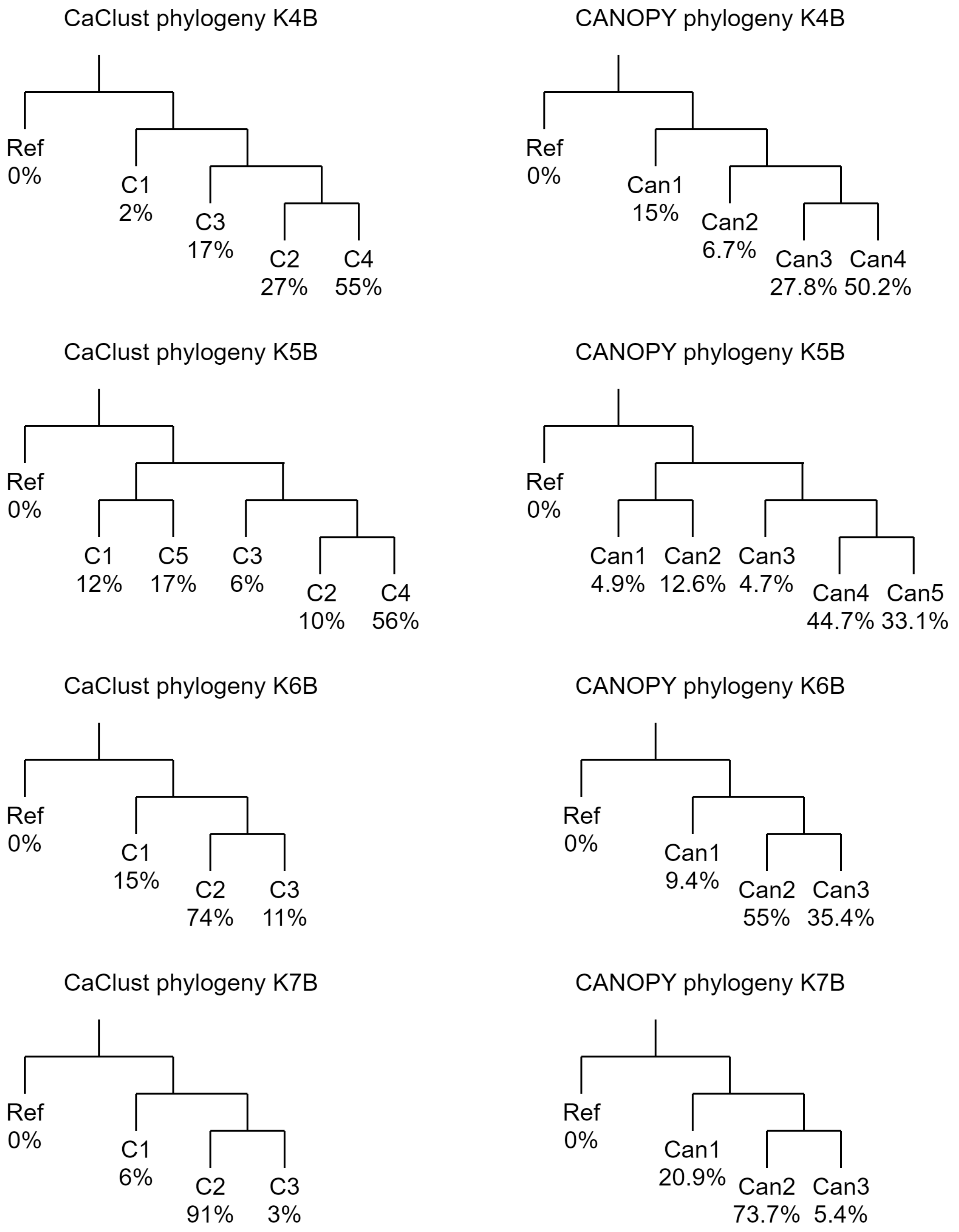

Fig. S13: Phylogenies obtained from hierarchical clustering of CaClust genotypes compared with input CANOPY phylogenies. Clones are not shared betweem samples. *Ref* - reference clone with no mutations, *C#* - indexed output CaClust tumour clone, *Can#* - indexed input CANOPY clone.

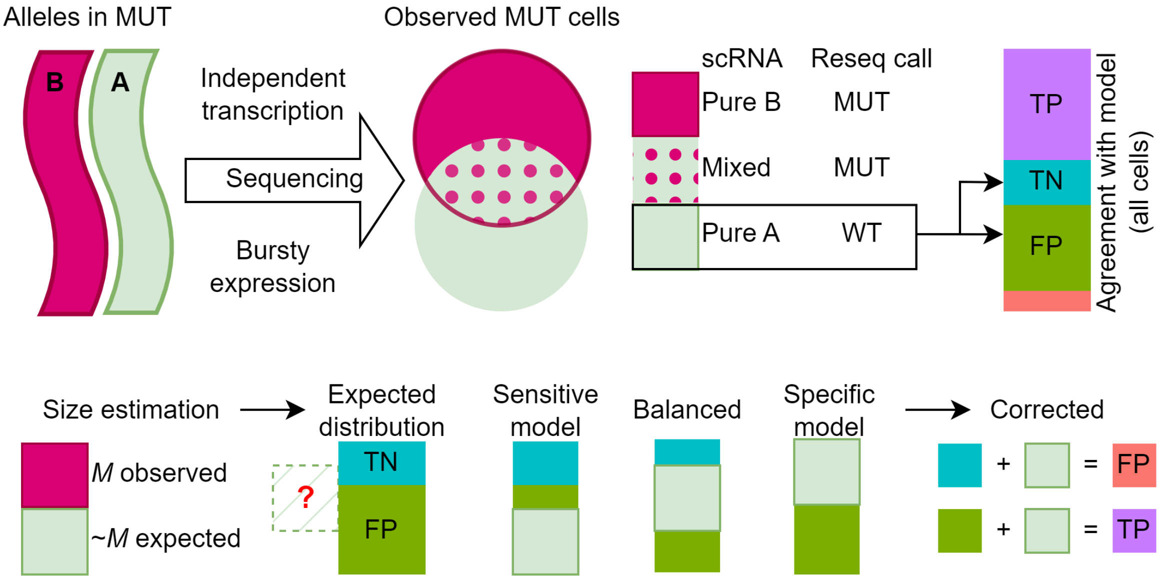

Fig. S14: Top: illustration of the effect of stochastic expression of heterozygotic mutations on resequencing calling; heterozygous mutant cells with only reference scRNA reads in resequencing are falsely classified as wildtype, inflating the True Negative (TN) and False Positive (FP) agreement with calls from other methods. **Bottom**: Random monoallelic correction application. The effect size is estimated based on the number of cells with only mutant scRNA reads of the mutated position. The true distribution of the falsely classified cells between the TN and FP agreement calls is unknown and depends partially on the model's sensitivity, to avoid bias we chose to apply a balanced correction in the paper.
