# Supplementary material for: CaClust: linking genotype to transcriptional heterogeneity of follicular lymphoma using BCR and exomic variants
Source: Genome Biol. 2024 Nov 5;25:286. doi: 10.1186/s13059-024-03417-1 (PMC11536712; doi:10.1186/s13059-024-03417-1)
Supplement: Supplementary file 2 — Additional file 2: Supplementary tables S1-S4 and supplementary notes on targeted resequencing and a correction to account for random monoallelic expression [58–61]. [file 13059_2024_3417_MOESM2_ESM.zip › 13059_2024_3417_MOESM2_ESM.pdf]

Supplementary Notes

Model validation with targeted resequencing

As an additional validation of CaClust results, we performed an independent resequencing experiment, targeted for the variant sites in single cells (Methods). For samples K6B and K7B, 8 variant genes used in the model inference were included in single cell resequencing: *FCHSD2*, *TNFRSF14*, and *MFHAS1* from K6B; *AFF2*, *ANKRD12*, *CLSTN3*, *DNAJC11*, and *VMA21* from K7B. Their genotypes obtained from resequencing were compared with the the results of cell genotyping by CaClust.

However, in the resequencing results a high proportion of mutant cells showed monoallelic variant expression, as measured by the fraction of mutant cells with only mutant UMIs of a variant and no corresponding reference UMIs (pure mutant fraction, Table S2). Whether this is a result of the stochastic nature of gene transcription or the technical limitations of scRNA sequencing, in which not all transcripts are observed, it is a sign of a problem with cell genotyping based purely on the targeted resequencing. Specifically, for a given heterozygous variant, we expect the same number of cells with exclusively monoallelic alternative reads, as the number of cells with monoallelic reference reads for this variant, which would falsely classify them as wildtype. To account for this we proposed a simple correction for such random monoallelic expression (see next section and Fig. S14). In short, it modifies the total number of wildtype and mutant calls in resequencing to account for the possible misclassfied mutants. Importantly, the correction is agnostic to their CaClust genotypes to avoid bias (see next section). We applied the correction to heterozygous variants that showed any monoallelic variant expression in the resequencing data: *FCHSD2*, *TNFRSF14*, *CLSTN3*, *ANKRD12*, *DNAJC11*. Additionally, it must be noted that in the *VMA21* and *AFF2* homozygous mutations we also observed cells with a mixture of variant and mutated reads, which shows the inherent noise in sequencing experiments with amplification stages that could affect the agreement results.

We found that the VAF estimation from the corrected numbers of genotype calls in resequencing was in better agreement with the true VAF obtained from WES (Table S2). The VAF estimation from the resequencing results undervaluated the true VAF in all 8 variants, which hints at mutant cells being missed in initial resequencing calling. The correction application improved the VAF estimation for all variants, which stands as motivation behind its use, although in the case of a single gene *DNAJC11* it did result in an overvaluation of the VAF.

We calculated the agreement between resequencing and CaClust genotypes as the fraction of cells with the same genotype call on a variant position (mutated or wildtype). Despite the aforementioned inherent noisy nature of the scRNA sequencing data, CaClust achieved very high ($> 90\%$) agreement for all 3 variants that did not qualify for the correction (homozygous *AFF2*, *VMA21*, or *MFHAS1* with no resequenced mutant reads). For the 5 heterozygous variants with possible missed mutant calls in the resequencing, we found 1 variant to be in high agreement before the correction, and 3 variants to be in high agreement after the correction (Table S2). Altogether, this shows the applicability of CaClust's use for single-cell genotyping in FL and stands as additional validation for our model.

Random monoallelic observation correction

In the analysis of the agreement between the model results and the resequencing genotype calling we apply a correction for the possibility of random monoallelic observations of the reference allele at heterozygous mutation positions, that accounts for dynamic autosomal random monoallelic expression (dynamic aRME) and the technical dropout of the sequencing experiments.

Maternal and paternal copies of genes are transcribed independently and in short intense bursts of transcription. Studies on allele-specific RNA transcription [58–61] have shown varying levels of RNA from maternal and paternal alleles within cell populations, with frequent observations of RNA from only one allele in a cell at a single point in time. In contrast to inherited aRME (clonal), this dynamic aRME is due to the stochastic nature of the gene expression process and is widespread across cell populations [58–60].

In our data we also observe monoallelic expression, both in the scRNA input data and the resequencing experiment, where a fraction of cells exhibits only variant reads of a heterozygous mutation, rather than a mix of variant and reference reads (with no CNA in that mutation's region). A converse effect is therefore expected, in which only the reference allele was observed for a number of cells carrying the mutation. That poses a problem, since those cells would be misclassified in resequencing as non-carriers, thus inflating the False Positive (FP) and possibly True Negative (TN) numbers in mutation agreement between resequencing and CaClust.

As a solution we propose a simple correction for this random monoallelic observation (Fig. S14). Since the cells with a heterozygotic mutation harbour one copy of each of the alleles and each copy is expressed independently, we expect there to be a balance: if M cells are observed with purely mutated reads and N cells are observed with mixed variant and reference reads, we statistically expect further M mutated cells to have expressed only the reference allele and be misclassified by the resequencing genotype calling.

Of course, the distribution of those misclassifications between the FP and TN calls in agreement is not obvious; therefore, we distribute them proportionally.

The theoretical description of the correction is as follows. Let M be the number of cells in which only the mutated reads are observed and let TP, FP, TN, FN be the numbers of true positive, false positive, true negative and false negative initial calls in the agreement between resequencing and CaClust. Then we obtain the corrected agreement numbers TP', FP', TN', FN' as described in Table S3.
