# Supplementary material for: CaClust: linking genotype to transcriptional heterogeneity of follicular lymphoma using BCR and exomic variants
Source: Genome Biol. 2024 Nov 5;25:286. doi: 10.1186/s13059-024-03417-1 (PMC11536712; doi:10.1186/s13059-024-03417-1)
Supplement: Supplementary file 2 — Additional file 2: Supplementary tables S1-S4 and supplementary notes on targeted resequencing and a correction to account for random monoallelic expression [58–61]. [file 13059_2024_3417_MOESM2_ESM.zip › Supplementary_Tables.pdf]

Supplementary Tables

Sample	Variant	Agreement (%) before	Agreement (%) after	Pure mutant fraction	VAF relative error before	VAF relative error after
K6B	FCHSD2	30	41	0.74	-0.45	-0.04
	TNFRSF14	92	91	0.47	-0.80	-0.70
	MFHAS1	100	100	⋆	⋆	⋆
K7B	CLSTN3	54	100	0.83	-0.46	0.00
	ANKRD12	32	42	0.60	-0.50	-0.20
	DNAJC11	50	90	0.89	-0.33	0.27
	AFF2	97	○	0.95	○	○
	VMA21	98	○	0.73	○	○

Table S1: Agreement of CaClust and resequencing results before and after the correction for random monoallelic expression. Pure mutant fraction is the ratio of cells with only mutant UMIs to cells with any mutant UMIS in the resequencing. VAF relative error is calculated as the relative difference between the true VAF from WES and the VAF predicted with resequencing genotypes before or after the correction. ⋆ – no variant *MFHAS1* reads were detected in resequencing; ○ – the correction does not apply to monoallelic *AFF2* and *VMA21*.

	if $M \leq (FP+TN)$	if $M > (FP+TN)$
TP'	$TP + \frac{FP}{FP+TN} \cdot M$	$TP + FP$
FP'	$FP - \frac{FP}{FP+TN} \cdot M$	0
TN'	$TN - \frac{TN}{FP+TN} \cdot M$	0
FN'	$FN + \frac{TN}{FP+TN} \cdot M$	$FN + TN$

Table S2: Application of a correction for random monoallelic expression with a balanced distribution.

	G1	S	G2M	
C1	365	17	10	392
C2	5011	513	341	5865
C3	130	14	23	167
	5506	544	374	

Table S3: Cell cycle distribution of clones in sample K7B.

Simulation scenario	μ_D	α_0	s_g	r_{clust}	r_{cell}
Basic	0.01	5	0.01	0	0
High reads	0.1				
Low reads	0.001				
Sparse hyperclustering		50			
High variance BCR			0.1		
80% centroids in 80% of hyperclusters				0.8	0.8
80% centroids in 20% of hyperclusters				0.8	0.2
20% centroids in 80% of hyperclusters				0.2	0.8
20% centroids in 20% of hyperclusters				0.2	0.2

Table S4: Simulation scenarios with their specific parameters. Parameters in blank spaces are the same as in the basic scenario.
