# Supplementary material for: CaClust: linking genotype to transcriptional heterogeneity of follicular lymphoma using BCR and exomic variants
Source: Genome Biol. 2024 Nov 5;25:286. doi: 10.1186/s13059-024-03417-1 (PMC11536712; doi:10.1186/s13059-024-03417-1)
Supplement: Supplementary file 5 — Additional file 5: Review history. [file 13059_2024_3417_MOESM5_ESM.docx]

**1^st^ round**

**Reviewer 1**

This paper develops a new method, CaClust, for analyzing interplay of clonal evolution and expression variability in B-cell lymphomas and applies it to examining data from several cases of follicular lymphoma. The method at a high level takes advantage of B-cell receptor sequencing to provide a more precise clustering of tumor cells than is typically possible in cancer clonal evolution inference to make accurate clonal assignments. This capability is integrated into a Bayesian graphical model for inference of the tumor clonal structure, mutation and expression profiles, and their dependencies. The model is impressively well thought out and engineered, with a detailed mathematical description and justification and excellent attention to challenges of model inference. The work is compared to the group's prior method, CACTUS, as well as to ablated variants of the methods, showing substantial improvements in accuracy on simulated data under diverse parameter regimes and in measures of confidence and plausibility of assignments on real data. Analysis of real data cases provides a good demonstration of value of the methods, as well as novel insight into clonal structure of these tumors and the degree to which genetic evolution can (or cannot) explain expression variability. There is also potential translational value in better identifying effective therapeutics for precision medicine and perhaps in identifying new drug targets and diagnostics. This is a technically interesting and rigorous work with potential value to those studying B-cell cancers and to computational methods developers in the field. It does, however, raise some questions for me:

1. One very general difficulty in evaluating this work is assessing the significance of the contribution. There is a great deal of study of clonal evolution in cancers and although this method is a clever improvement on other work in this domain, it is for a fairly specialized application for the field specific to this kind of B-cell lymphoma. That is of medical importance, although the authors might make the case more strongly for need for new methods for the specific disease. For methods developers, though, think this would be seen as a fairly narrow application in itself, and I would wonder if there is any broader potential application of the techniques developed here. Would the principles behind it apply to other cancers than B-cell lymphomas, or example, or other sources of data informative of cell clustering than B-cell receptor sequencing?

2. Again on the question of significance, I think the authors could make a stronger case for the improvement relative to CACTUS. It is appropriate that the authors compare to CACTUS, since it seems to be the only competing method for this task. There is clearly an advance, in the methodology and the results. But it is not clear that this is more than an incremental advance. I would suggest making a more persuasive case specifically for the computational innovation relative to the prior work and why the improved results are more than an incremental advance.

3. The paper does an impressive job of following up on the likely biological basis in those cases for which expression variation can be clearly tied to genetic evolution. However, I feel it falls short in explaining the cases where expression variation does not correspond well to clonal evolution. Where somatic genetic variation does not account for expression variation, then what is? I realize the authors cannot reasonably be expected to explore every possible cause, but I wonder if there are obvious ones that could be explored. For example, is a large portion of expression variation mostly accounted for by cell cycle variation, or by variations in microenvironment? It would seem that one could at least ask which genes account for most of the expression variation and whether prior knowledge or a term enrichment analysis links these to any specific biological processes.

4. A related question is whether there is genuine genetically-driven expression variation that is being washed out by larger sources of non-genetic variation. That is, if one subtracts some of the major non-genetically-driven sources of expression variation, such as cell cycle changes, do genetically-driven expression changes then emerge for clones that did not previously show them? If one looks at mutated driver genes of clones that do not show genetically-driven expression variation, can one find genetically-driven expression changes with a more targeted search for genes in common pathways with those driver genes?

5. The paper does a nice job of drawing conclusions about the biology from the results on real cases, but these final conclusions are somewhat unsatisfying and I think it is largely a consequence of a very small sample size. Essentially, the four cases are each quite different, and there is no consistent trend among clones within the cases; some clones in some patients show expression phenotype following genotype, and the rest do not. While that is a useful observation, I would expect that with a larger cohort size, trends would start to emerge across subsets of patients and subsets of clones. Are there independent data sources to which one might apply CaClust to pull out some of these trends (which would be useful anyway in validating the method)? If there is no such data, I think that needs some comment and at least a brief discussion of what it would take to do an adequately powered study with CaClust.

6. I noted a couple more very minor points:

- line 38: "to which extent" should be "to what extent"

- line 58 makes an assertion that it is not possible to look at single-cell DNA and RNA in the same cells. There is at least one commercial outfit offering a technology to do this (Bioskryb's ResolveOME platform) and perhaps others of which I am unaware..

**Reviewer 2**

The authors investigate the relationship between genomic and transcriptional heterogeneity in a dataset of follicular lymphoma (FL), by means of a probabilistic model that integrates bulk whole-exome, single-cell RNA and B-cell receptor (BCR) sequencing data. Their results are supported by both in-silico data generated for the purpose of this study, and new high-quality real data. They also provide a validation dataset of targeted single-cell sequencing.

The model is well documented and the authors evaluate its performance through extensive simulations resembling varying scenarios. Although the results presented in the paper are very interesting and address an important open question of cancer evolution through a novel methodology, I have some concerns, especially regarding the interpretation of the results.

Major

- In all the real world cases presented in the Results section, it is not clear what is the phylogenetic tree underlying the inferred clonal architecture. The authors use CANOPY to deconvolve clones from WES data, and this tool produces a phylogeny that is input to their model. Then the clone genotypes, assumed by the authors to be affected by noise with a parameterized error rate, are corrected based on the integration of single-cell data. I found it extremely difficult to reconstruct a phylogeny from the finally proposed clones from their genotypes. For example, the genotypes inferred for sample K7B (Figure 4) present a set of mutations (DPYSL2 and MRPS5) that are shared between clones C1 and C2 but not with C3, suggesting a most recent common ancestor for C1 and C2 to be put on a different tree branch with respect to C3. Conversely, another set of mutations (HMGN1, RALGAPA2 and PLEKHA4) shared between C1 and C3 but not with C2, suggest a different tree in which C2 sits on a different branch with respect to the most recent common ancestor of C1 and C3.

In my opinion, the authors should provide a more precise interpretation of the clonal architecture of each sample, and discuss the results of gene expression analysis in the light of the inferred phylogenetic tree.

- Another important case is that of the longitudinal assay involving samples K4B and K5B. This case study is particularly interesting because the authors claim to observe selection acting on one of the inferred clones. Again, I found it hard to interpret the phylogenetic relationship between the inferred clones from the proposed genotypes. Here in particular, I think it would be useful to present the results of the multi-sample clonal deconvolution provided by Canopy, as this should result in clusters of private and shared mutations across the two subsequent samples, clarifying the underlying phylogenetic tree.

- Generally, in addition to the number of clones, subclonal deconvolution methodologies estimate the relative fraction of each clone. Even after genotype correction, is it possible to determine the degree of consistency between the fractions estimated from bulk data and the number of single cells assigned to each clone? I think the overall interpretation of data would benefit from this type of analysis.

- Regarding the interpretation of the gene expression analysis for sample K7B, the authors draw important conclusions regarding the effects of clone genotypes on the different fractions of cells across cell-cycle phases. It seems to me that this analysis suffers from a very low number of cells at play (< 15% clone fraction in a relatively small clone). I invite the authors to explicitly report abundance of cells and comment.

Minor

The github repository containing the code is poorly documented. Given the potential of CaClust in the joint analysis of genomic and transcriptional heterogeneity, it would be useful to make the code available to the community and better documented for sake of user-friendliness and reproducibility of the results.

**Authors’ response**

Reviewer #1

1. One very general difficulty in evaluating this work is assessing the significance of the contribution. There is a great deal of study of clonal evolution in cancers and although this method is a clever improvement on other work in this domain, it is for a fairly specialized application for the field specific to this kind of B-cell lymphoma. That is of medical importance, although the authors might make the case more strongly for need for new methods for the specific disease.

For methods developers, though, think this would be seen as a fairly narrow application in itself, and I would wonder if there is any broader potential application of the techniques developed here. Would the principles behind it apply to other cancers than B-cell lymphomas, or example, or other sources of data informative of cell clustering than B-cell receptor sequencing?

Thank you for this comment. We now make a stronger statement of the need for dedicated methods for analyzing follicular lymphoma evolution in the Background section (page 3). Indeed, the model was designed specifically for the application to follicular lymphoma, where the evolutionary process takes place on the exome level and in parallel, on the level of BCR sequences.

However, the model could easily be applied with simple modifications to any other use cases where additional hyperclusters of cells belonging to the same clone could be identified. We now mention this in the Discussion section (page 18).

2. Again on the question of significance, I think the authors could make a stronger case for the improvement relative to CACTUS. It is appropriate that the authors compare to CACTUS, since it seems to be the only competing method for this task. There is clearly an advance, in the methodology and the results. But it is not clear that this is more than an incremental advance. I would suggest making a more persuasive case specifically for the computational innovation relative to the prior work and why the improved results are more than an incremental advance.

Thank you for this comment! The improvement of CACTUS has been demonstrated on simulated data, clearly indicating higher performance of CaClust. The methodological advancement, coming from in-depth modeling of distributions of BCR sequences in the hyperclusters and from non-parametric Bayesian clustering approach, has practical benefits. In particular, CaClust achieves much higher confidence of assignment of single cells to their respective clones. Following the suggestion of the Reviewer, in this revision, to better showcase how the improvement in CaClust performance is crucial for the analysis of real data, we added the section “Higher confidence assignment of cells to clones by CaClust facilitates more informative downstream analysis as compared to CACTUS” and added Supplementary Figures 9-12. In particular, we show that cells assigned to different clones by CACTUS are more mixed in terms of gene expression (Supplementary Figure 9), show fewer differentially expressed genes with smaller statistical significance (Supplementary Figure 10), the CACTUS-identified clones do not show significant differences in cell cycle distributions as found for clones identified by CaClust (Supplementary Figure 11), and finally, that the noise may completely wash-out the expected clonal structure (Supplementary Figure 12). These comparisons demonstrated that due to more confident assignments of cells to clones, CaClust is able to deliver more biological findings and clearer conclusions from the data than CACTUS.

3. The paper does an impressive job of following up on the likely biological basis in those cases for which expression variation can be clearly tied to genetic evolution. However, I feel it falls short in explaining the cases where expression variation does not correspond well to clonal evolution. Where somatic genetic variation does not account for expression variation, then what is? I realize the authors cannot reasonably be expected to explore every possible cause, but I wonder if there are obvious ones that could be explored. For example, is a large portion of expression variation mostly accounted for by cell cycle variation, or by variations in microenvironment? It would seem that one could at least ask which genes account for most of the expression variation and whether prior knowledge or a term enrichment analysis links these to any specific biological processes.

We are grateful for this suggestion. To look into the other possible causes of expression variation we investigated the GO term enrichment in top 250 highly variable genes for samples K5B and K6B, finding factors associated with inherent B-cell functions. The new results are presented in section “FL samples show different strengths of genotype-phenotype influence” in paragraph 6 on page 9 and Supplementary Figures 4-5. Indeed, there could be other potential causes of variation that are out of scope of our work, and these are also mentioned in the same paragraph.

4. A related question is whether there is genuine genetically-driven expression variation that is being washed out by larger sources of non-genetic variation. That is, if one subtracts some of the major non-genetically-driven sources of expression variation, such as cell cycle changes, do genetically-driven expression changes then emerge for clones that did not previously show them? If one looks at mutated driver genes of clones that do not show genetically-driven expression variation, can one find genetically-driven expression changes with a more targeted search for genes in common pathways with those driver genes?

This is a very valid remark, and to investigate it we performed additional analyses restricted to cells from phase G1 of the cell cycle in all samples, finding that overall expression structure was preserved as compared to the full analysis. We include and comment on these results in section “FL samples show different strengths of genotype-phenotype influence” in paragraph 5 on page 8 and Supplementary Figure 3. We also performed the targeted search of possibly affected pathways, but found no additional enrichment.

5. The paper does a nice job of drawing conclusions about the biology from the results on real cases, but these final conclusions are somewhat unsatisfying and I think it is largely a consequence of a very small sample size. Essentially, the four cases are each quite different, and there is no consistent trend among clones within the cases; some clones in some patients show expression phenotype following genotype, and the rest do not. While that is a useful observation, I would expect that with a larger cohort size, trends would start to emerge across subsets of patients and subsets of clones. Are there independent data sources to which one might apply CaClust to pull out some of these trends (which would be useful anyway in validating the method)? If there is no such data, I think that needs some comment and at least a brief discussion of what it would take to do an adequately powered study with CaClust.

We recognise that the sample size included in the analysis is relatively small. We searched for independent datasets that could be used in our model (NCBI Gene Expression Omnibus, EGA Genome-Phenome Archive, Chan Zuckerberg Initiative); however, as the model requires a very specific combination of data sources (WES, scRNA, scBCR) we were not able to find one that would contain all three of them. We expect that the existence of our method, enabling us to draw reliable, new biological conclusions from a combination of established experimental methods will encourage other labs to generate similar data, enabling analysis of larger cohorts. This consideration is now mentioned in the Discussion section (page 18).

6. I noted a couple more very minor points:

- line 38: "to which extent" should be "to what extent"

Thank you for diligence, the mistake has been corrected.

- line 58 makes an assertion that it is not possible to look at single-cell DNA and RNA in the same cells. There is at least one commercial outfit offering a technology to do this (Bioskryb's ResolveOME platform) and perhaps others of which I am unaware.

Thank you for pointing this out. We modified the Background and now mention that platform, which suffers from limited throughput. .

Reviewer #2

1. In all the real world cases presented in the Results section, it is not clear what is the phylogenetic tree underlying the inferred clonal architecture. The authors use CANOPY to deconvolve clones from WES data, and this tool produces a phylogeny that is input to their model. Then the clone genotypes, assumed by the authors to be affected by noise with a parameterized error rate, are corrected based on the integration of single-cell data. I found it extremely difficult to reconstruct a phylogeny from the finally proposed clones from their genotypes. For example, the genotypes inferred for sample K7B (Figure 4) present a set of mutations (DPYSL2 and MRPS5) that are shared between clones C1 and C2 but not with C3, suggesting a most recent common ancestor for C1 and C2 to be put on a different tree branch with respect to C3. Conversely, another set of mutations (HMGN1, RALGAPA2 and PLEKHA4) shared between C1 and C3 but not with C2, suggest a different tree in which C2 sits on a different branch with respect to the most recent common ancestor of C1 and C3. In my opinion, the authors should provide a more precise interpretation of the clonal architecture of each sample, and discuss the results of gene expression analysis in the light of the inferred phylogenetic tree.

Thank you for this comment. Indeed, the current genotype correction in CaClust is not enforced to maintain the tree structure of the clones, which we recognise is a limitation. However, to be able to correct clone genotypes in our model in a realistic manner that respects the phylogeny, the model would have to account for events other than single nucleotide mutations, such as insertions, deletions, sequencing depth and amplification. While theoretically possible, such a model extension would require a significant theoretical development which constitutes future work. We now mention these prospects in the Discussion section on page 18.

To address the concerns of the Reviewer and to investigate the most likely phylogenies behind the clones in the analyzed samples, we performed hierarchical clustering of the inferred (corrected) clone genotypes. Although the genotypes were changed from the input ones originally inferred by Canopy, the resulting structure of the phylogenies remained unchanged in all cases. These new results are featured in a new section “Analysis of CaClust clonal genotypes shows agreement with input phylogenies” and Supplementary Figure 13. The new section also features an investigation of the possible sources of disagreement with the input phylogenies, which include the low read count on some positions, as well as the occurrence of deletions.

2. Another important case is that of the longitudinal assay involving samples K4B and K5B. This case study is particularly interesting because the authors claim to observe selection acting on one of the inferred clones. Again, I found it hard to interpret the phylogenetic relationship between the inferred clones from the proposed genotypes. Here in particular, I think it would be useful to present the results of the multi-sample clonal deconvolution provided by Canopy, as this should result in clusters of private and shared mutations across the two subsequent samples, clarifying the underlying phylogenetic tree.

This is a very interesting remark, which we address in paragraph 6 on page 15 added to the Results section on samples K4B and K5B. As demonstrated in the new Supplementary Figures 11-12, the multisample CANOPY deconvolution of WES allele frequencies was unable to provide a phylogeny that would show the expected structure of shared mutations between the samples higher up the tree and clones with sample specific mutation as leaves. This highlights the difficulties of estimating the clonal composition of the samples based on bulk data alone and was the reason we were unable to use the Canopy model on the joined samples. Still, with the use of CaClust, we were able to elucidate the evolutionary history of the samples by using the input from Canopy on individual samples.

3. Generally, in addition to the number of clones, subclonal deconvolution methodologies estimate the relative fraction of each clone. Even after genotype correction, is it possible to determine the degree of consistency between the fractions estimated from bulk data and the number of single cells assigned to each clone? I think the overall interpretation of data would benefit from this type of analysis.

This is a very good note, as the degree of consistency between the estimates of the clonal fractions and the output frequencies of clones tie in to how much the approximate clonal structure from WES was changed by the scRNA observations. As discussed in the new section “Analysis of CaClust clonal genotypes shows agreement with input phylogenies” and visualized in Supplementary Figure 13, the frequencies of the corrected genotypes changed only moderately as compared to the frequencies estimated by Canopy.

4. Regarding the interpretation of the gene expression analysis for sample K7B, the authors draw important conclusions regarding the effects of clone genotypes on the different fractions of cells across cell-cycle phases. It seems to me that this analysis suffers from a very low number of cells at play (< 15\% clone fraction in a relatively small clone). I invite the authors to explicitly report abundance of cells and comment.

We strongly agree that special care has to be taken when analysing small clones. The clone populations are included as part of Figure 3, the cell counts for the clones are explicitly mentioned for K7B in the text (page 11), and we added Table 4 containing the clone cell counts across phases. To ensure statistical significance we followed the recommendations for the Pearson’s chi-2 independence test, where no entry in the counts table should have an expected value smaller than 5. As in our case the smallest expected value is 10.1, the obtained p-values are accurate and the conclusion that the clones differ in their cycle distributions is justified. We added this justification in the section “Cell cycle analysis” in the Methods.

5. Minor: The github repository containing the code is poorly documented. Given the potential of CaClust in the joint analysis of genomic and transcriptional heterogeneity, it would be useful to make the code available to the community and better documented for sake of user-friendliness and reproducibility of the results.

Thank you for pointing out the need for making CaClust more approachable, we added a README with descriptions of all files in the project, with two examples of running the model on toy and real data, which are also included. The main inference function also has the description of all required input and parameters, as well as the checkpoint functionality.

**2^nd^ round**

**Reviewer 1**

I appreciate the detailed response and many additional analyses the authors have done in response to the critiques. I believe all of my concerns about the original manuscript have been adequately addressed and I have no new concerns to raise.

**Reviewer 2**

The authors have addressed our comments. In particular

- The authors have introduced a new section in the paper where they extensively address our concerns. We suggest adding genomic position coverage to Figure 4a for a more comprehensive view of the data.

- In response to our comments, the authors have added a subsection in the main paper along with a new supplementary figure. We appreciate their explanation of why the multi-sample CANOPY deconvolution does not show the expected phylogeny.

- The authors have included the requested information in the new section of the paper and in a supplementary figure, briefly discussing its significance.

- The authors followed our suggestion by adding Table 4 where they report relative clonal abundance and perform a statistical test to assess where the counts are statistically significant.

- The additions to the GitHub repository enhance both the reproducibility and the overall understanding of the results.

**Authors’ response**

Regarding Reviewer 2 suggestion of adding coverages to Figure 4a, we decided not to

do this, as the minimum coverage present in each site is already mentioned in the text

and Additional File 1, and the actual values put into the matrix would blur the message

of the figure
